# Genomic analysis of *Luteimonas abyssi* XH031^T^: insights into its adaption to the subseafloor environment of South Pacific Gyre and ecological role in biogeochemical cycle

**DOI:** 10.1186/s12864-015-2326-2

**Published:** 2015-12-21

**Authors:** Li Zhang, Xiaolei Wang, Min Yu, Yanlu Qiao, Xiao-Hua Zhang

**Affiliations:** College of Marine Life Sciences, Ocean University of China, 5 Yushan Road, Qingdao, 266003 China; College of Life Science, Qingdao Agriculture University, Qingdao, 266109 China

**Keywords:** *Luteimonas abyssi*, Genomic analysis, Genetic advantages, Ecological role

## Abstract

**Background:**

*Luteimonas abyssi* XH031^T^, which was previously isolated from subseafloor environment of the South Pacific Gyre (SPG), was an aerobic, gram-negative bacterium, and was identified to be a novel species of the genus *Luteimonas* in the family of *Xanthomonadaceae*. The nutrients utilization and metabolic mechanisms of XH031^T^ indicate its plasticity. In view of the above characteristics, its genome was sequenced, and an in-depth analysis of the XH031^T^ genome was performed to elucidate its adaption to extreme ecological environment.

**Results:**

Various macromolecules including polysaccharide, protein, lipid and DNA could be degraded at low temperature by XH031^T^ under laboratory conditions, and its degradation abilities to starch, gelatin and casein were considerably strong. Genome sequence analysis indicated that XH031^T^ possesses extensive enzyme-encoding genes compared with four other *Luteimonas* strains. In addition, intricate systems (such as two-component regulatory systems, secretion systems, etc.), which are often used by bacteria to modulate the interactions of bacteria with their environments, were predicted in the genome of XH031^T^. Genes encoding a choline-glycine betaine transporter and 99 extracellular peptidases featured with halophilicity were predicted in the genome, which might help the bacterium to adapt to the salty marine environment. Moreover, there were many gene clusters in the genome encoding ATP-binding cassette superfamily transporters, major facilitator superfamily transporters and cytochrome P450s that might function in the process of various substrate transportation and metabolisms. Furthermore, drug resistance genes harbored in the genome might signify that XH031^T^ has evolved hereditary adaptation to toxic environment. Finally, the annotation of metabolic pathways of the elements (such as carbon, nitrogen, sulfur, phosphor and iron) in the genome elucidated the degradation of organic matter in the deep sediment of the SPG.

**Conclusions:**

The genome analysis showed that XH031^T^ had genetic advantages to adapt to subseafloor environment. The material metabolism manifests that the strain may play an important ecological role in the biogeochemical cycle of the SPG, and various cold-adapted extracelluar enzymes produced by the strain may have significant value in application.

**Electronic supplementary material:**

The online version of this article (doi:10.1186/s12864-015-2326-2) contains supplementary material, which is available to authorized users.

## Background

Marine sediments, the extreme ecological environment characterized by low light intensity, low temperature, low oxygen concentration and high hydrostatic pressure [1], account for more than two-thirds surface area of the earth. Temperature, energy, and hydrostatic pressure may be the primary challenges facing the subseafloor microbes. Some extreme microbes such as psychrophiles and barophiles can grow at those conditions [2]. Microorganisms existing hundreds of meters below deep seafloor were first described by Parkes in 1994 [3], which motivated a coordinated, systematic investigation of the subseafloor biosphere. Distinct from organisms living in the “light” biosphere, which are supported by the sunlight energy, the energy of microbes living in the dark biosphere mainly comes from chemical reactions [4]. The energy flux and circulation of materials in the dark deep-sea biosphere have become a research frontier and the focus of attention. In addition, the unique geochemical features of deep-sea conditions indicate that subseafloor microbes might play significant roles in deep-sea biogeochemical cycles [5].

Subseafloor sediments have accumulated about 10 billion tons of organic matter [6]. As decomposer of deep-sea sediment, microbes are crucial in digesting organic matter by means of secreting extracellular hydrolytic enzymes. Most importantly, these enzymes, which are psychrophilic, display a high catalytic efficiency in biogeochemical processes and have high potentials in biotechnology and industry applications [7]. As additives and biocatalysts, psychrophilic enzymes have also been used in the process of chemical synthesis, that can not only minimize unwanted side reactions which happen at higher temperatures, but also preserve essential nutritional value and flavor of food in foodstuff industries [8]. However, very few researches have been conducted regarding the psychrophilic enzymes secreted by microbes from subseafloor sediments.

XH031^T^ was isolated from the depth of 18.1–18.2 m below sea floor sediment (5074 meters below sea level) of the South Pacific Gyre (SPG) at station U1370 (41.51° S 153. 6° W), where oxygen persists through the entire sediment sequence to depths of at least 75 meters below sea floor [9]. The strain belongs to *Gammaproteobacteria* and is a Gram-negative, strictly aerobic, yellow and rod-shaped bacterium [10]. The strain has been found to secrete various exoenzymes when it was identified as a novel species in our previous studies. Oxidase- and catalase- are positive in XH031^T^ and starch, gelatin, casein and Tween 20, 40 and 80 can also be digested by the strain. Additionally, esterase (C4), valine arylamidase, trypsin, α-chymotrypsin, α-glucosidase, leucine arylamidase, alkaline (and acid) phosphatase activities are present in this strain [10]. Meanwhile, some gene clusters might have been developed by the strain to adapt to the deep sediments. Genomic analysis of XH031^T^ would indicate how various nutrients are hydrolyzed and on what nutrients this strain depends to live in the extreme environment. Moreover, genome sequence data would be quite helpful in developing detailed hypothesis on the special role of *Xanthomonadaceae* members in marine biogeochemical cycling. Therefore, the whole genome of XH031^T^ was sequenced and analyzed, and the genomic comparison with other two bacteria in the genus of *Luteimonas* was also performed. The results provide the first picture of XH031^T^ in adaptation to the extreme environment of the subseafloor sediment.

## Results and discussion

### Characteristics of XH031^T^ and the abilities to digest various macromolecules

After incubating 2–3 days at 28 °C on marine agar 2216 (MA; Becton Dickinson), the strain formed circular (1.0–1.5 mm in diameter), yellow-pigmented, convex, and slightly transparent colonies. 16S rRNA gene sequence showed that it has 96.95 % similarity with *Luteimonas aestuarii* B9^T^, and the data from polyphasic analysis also indicated that the strain represents a novel species of the genus *Luteimonas* [10].

By using different culture media, at least four kinds of macromolecules could be degraded by XH031^T^ at low temperature under laboratory conditions. These macromolecules include polysaccharides (starch, cellulose and chitin), proteins (gelatin and casein), lipids (Tween 20, 40 and 80) and DNA. The strain had stronger enzymatic activities of amylase, gelatinase, cellulase and caseinase than those of DNase, lipase and chitinase. In the polysaccharide hydrolyase family, it held higher hydrolytic abilities to starch and cellulose than that of chitin. Meanwhile, the protease (including gelatinase and caseinase) activities were equally high with that of amylase. In addition, this bacterium showed catalase activity but no lecithinase activity (Table 1).

**Table 1:** Enzymatic activities detected in XH031^T^

Enzymes	Temperature		
	28°C	16°C	4°C
Amylase	++++^a^	++++	+++
Gelatinase	+++	++++	+
Cellulase	+++	+	+
Caseinase	+++	+++	W
DNase	+	+	–
Chitinase	+	+	–
Tween 20 Lipase	+	+	–
Tween 40 Lipase	+	+	–
Tween 80 Lipase	+	W	–
Lecithinase	–	–	–
Catalase	+	+	+

^a^W: Weak positive; +: Positive; +++ or ++++: Highly positive; –: Negative

Hydrolytic circle was used to detect enzymatic activities by the value of H/C. H: diameter of hydrolytic circle (cm); C: diameter of colony (cm). Weak (W): 0 < H/C ≤ 0.1; +: 0.1 < H/C ≤ 1.0; ++: 1.0 < H/C ≤ 2.0; +++: 2.0 < H/C ≤ 3.0; ++++: H/C > 3.0

### Global genomic characteristics and comparison with other *Luteimonas* genomes

The genome of XH031^T^ is composed of 3,988,191 bp (one chromosome with no plasmid) and the calculated G + C content is 69.26 %, which is slightly lower than the experimentally determined 70.2 % [10]. Six rRNA genes (including two 5S rRNAs, two 16S rRNAs and two 23S rRNAs) and 51 tRNA genes were identified in the genome. The number of tandem repeat sequence is 272 and the total length of tandem repeat sequence is 29,798 bp, which accounts for 0.75 % of the whole genome. In addition, 21 microsatellite DNA and 191 minisatellite DNA were found in the genome. The general genomic features of XH031^T^ were described in [Additional file 1: Table S1].

**Fig. 1:**
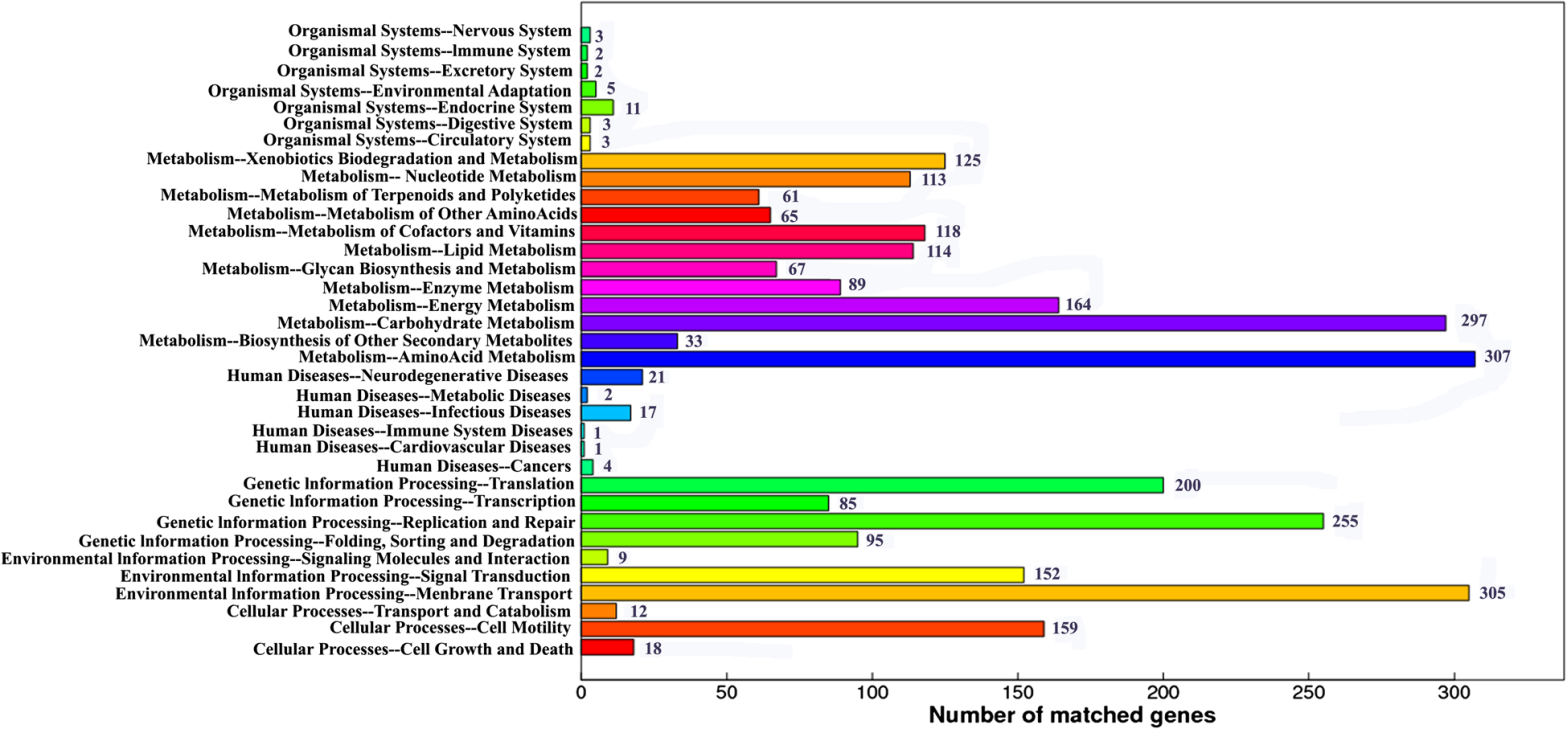
KEGG pathway classification of the genome of strain XH031^T^. Functional classification of XH031^T^ genome ORFs based on the KEGG database. In total, 2,918 ORFs had functional classifications assigned and the numbers with each classification are indicated

**Fig. 2:**
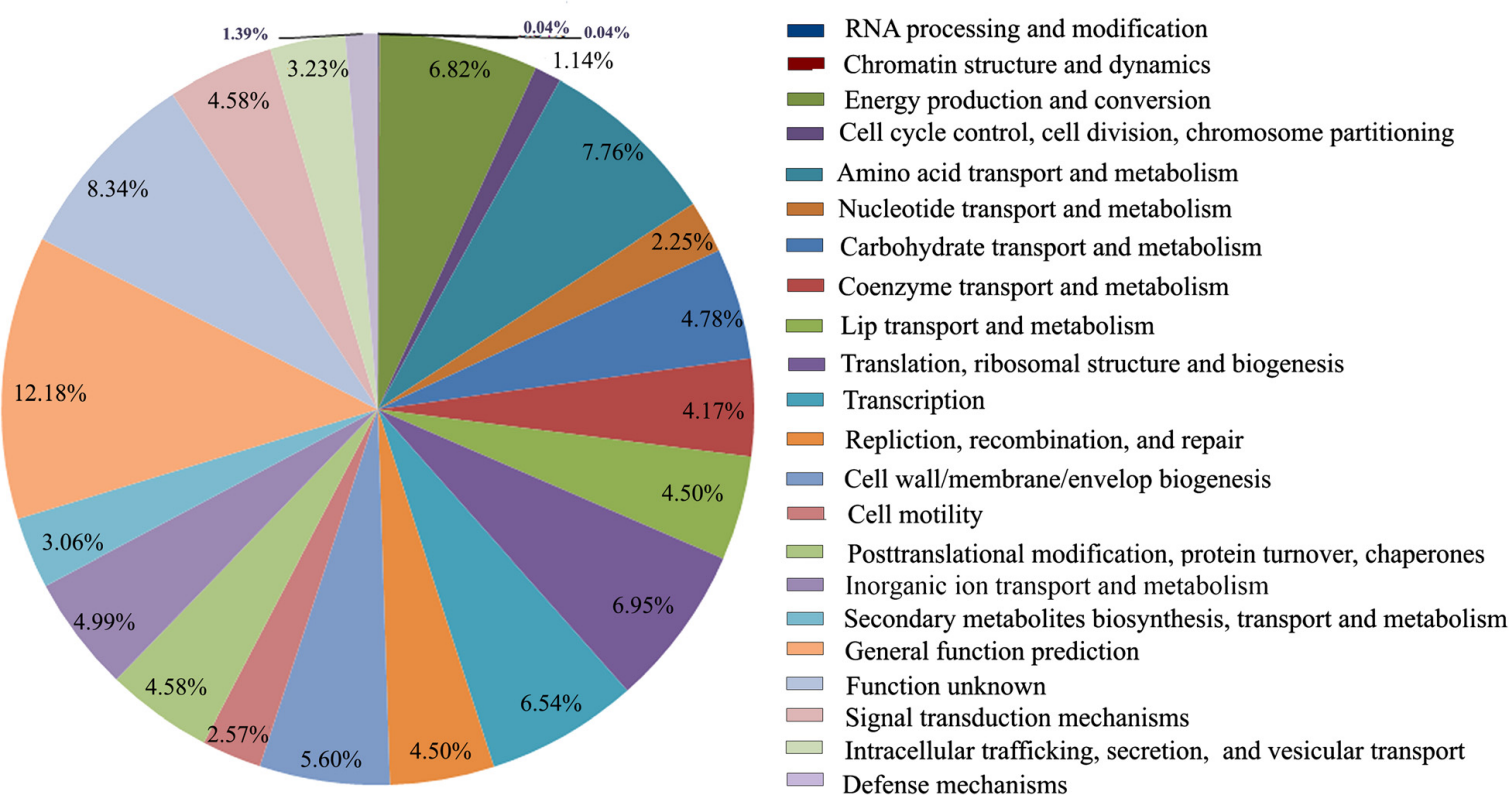
Functional classification of ORFs encoded by XH031^T^ genome based on the COG. In total, 3,951 ORFs with orthologs in the COG database were classified and the percentages indicate the frequencies of ORFs with assigned functions

A total of 3,605 coding sequences (CDSs) were predicted within the genome of XH031^T^. Among the predicted CDSs, 2,918 (80.9 %) genes were predicted in the Kyoto Encyclopedia of Genes and Genomes pathway (KEGG) database, 2,483 (68.9 %) genes were annotated in the Cluster of Orthologous Groups of proteins (COG) categories, while 3,056 (84.8 %), 1,418 (39.3 %) and 3,006 (83.4 %) genes were applicable within nonredundant (NR), SwissProt and TrEMBL databases, respectively. At the same time, 2,885 matched genes were predicted to be involved in 35 metabolic pathways predicted in the KEGG database. Eighty-nine (2.3 %) and 297 (7.5 %) matched genes are involved in encoding enzyme-families and carbohydrate metabolism pathways (Fig. 1), respectively. There were about 4.8 % genes involved in the process of carbohydrate transport and metabolism according to COG categories (Fig. 2). In addition to general function prediction only (R) and function unknown (S) genes, the largest proportion of orthologous genes with certain function in XH031^T^ were predicted to be related with amino acid transport and metabolism (7.8 %), which might enable the strain to degrade organic matter, especially for the decomposition of sedimentary organic nitrogen in the oligotrophic sediment.

**Fig. 3:**
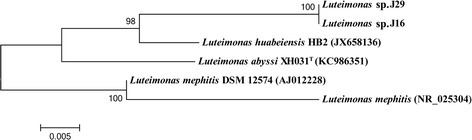
Phylogenetic tree of XH031^T^ and other *Luteimonas* strains based on 16S rRNA gene. Tree was produced by neighbor-joining method with 1000 bootstrap replications. The sequence alignment and phylogenetic calculations were performed with MEGA 5.0

The genome of XH031^T^ is larger than that of the three other *Luteimonas* strains: *L. mephitis* DSM 12574 (AULN01000000), *Luteimonas* sp. J29 (AWZR01000000), *Luteimonas* sp. J16, and is smaller than that of *L. huabeiensis* HB2 (JAAN01000000). *L. mephitis* DSM 12574 was isolated from experimental biofilters used for the waste gas treatment of an animal-rendering plant, while HB2 was isolated from water samples collected from stratum water located in Huabei Oilfield, China. Additionally, XH031^T^ harbored the largest number of tRNA, rRNA and COG clusters compared with four other strains. Basic features of these genomes were given in Table 2 and their phylogenetic relationship on the basis of 16S rRNA gene sequence was shown in Fig. 3. Phylogenetic tree indicated that XH031^T^ formed a tight phylogenetic cluster with *L. huabeiensis* HB2, while *Luteimonas* sp. J29 and J16 shared the same 16S rRNA gene sequence.

**Table 2:** Genome features of XH031^T^ and other *Luteimonas* genomes

	*L.abyssi* XH031^T^	*L.huabeiensis* HB2	*L.mephitis* DSM 12574	*Luteimonas *sp*.* J29	*Luteimonas* sp*.* J16
Genome size (bp)	3,988,191	4,295,921	3,416,011	3,401,613	3,419,099
G + C content (%)	69.26	71.6	68.5	71.9	71.76
CDS length	3,549,861	3,862,454	3,028,522	3,083,695	3,066,469
tRNA number	51	45	45	44	45
rRNA number	6	3	3	3	3
Gene number	3,605	3,778	2,658	2,667	3,170
COG cluster number	2,447	1,647	1,469	1,566	1,532

**Fig. 4:**
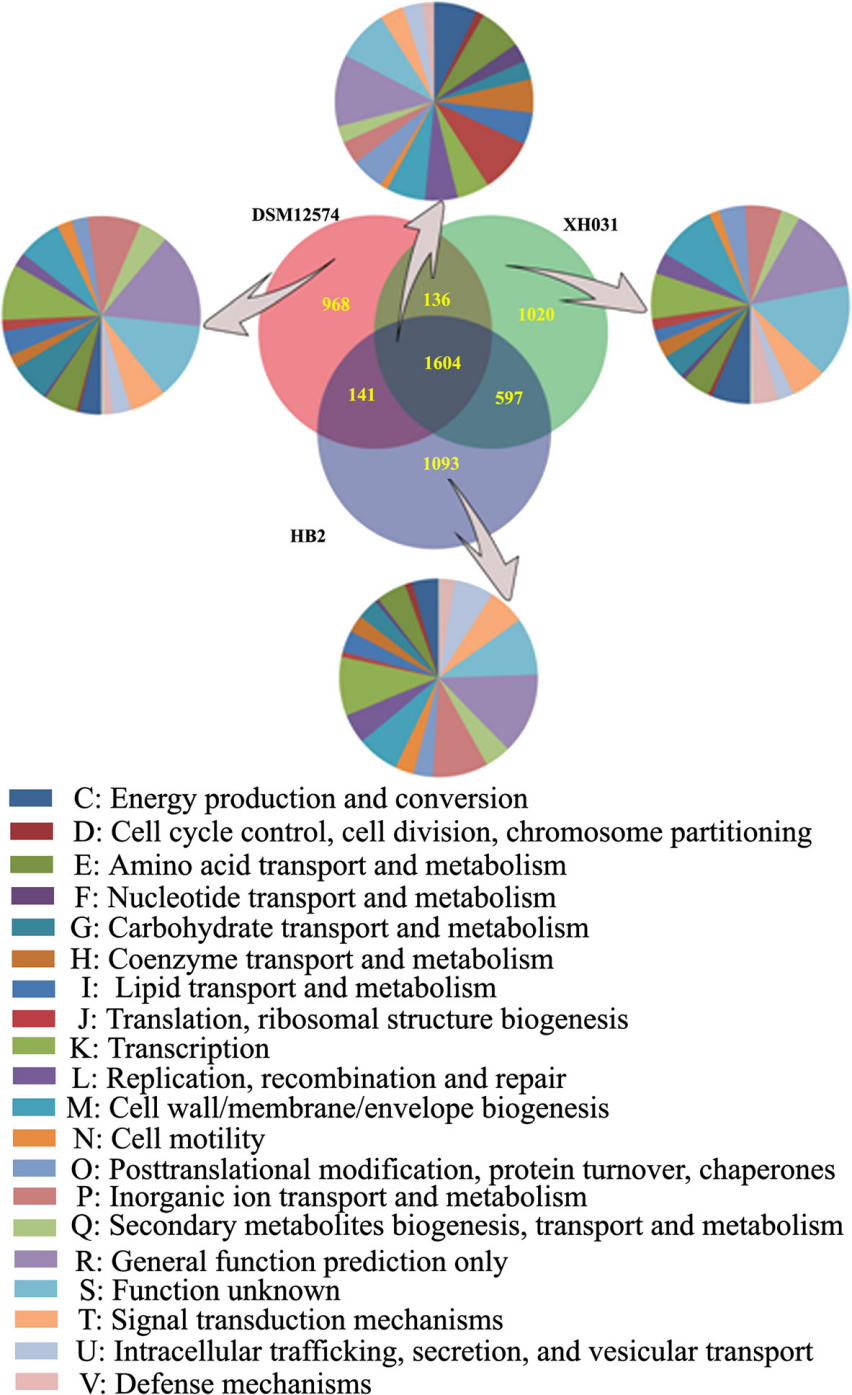
Comparison of specific and core genes among XH031^T^, DSM 12574 and HB2. The Venn diagram shows the number of orthologous and specific gene clusters among each strain, and the pie charts show the relative abundance compared to all COG categories of the orthologous and specific genes in each strain

Further comparison on the basis of all the predicted protein sequences of XH031^T^, DSM 12574 and HB2 were made (Fig. 4). Although these isolates belonged to different species of genera *Luteimonas*, they shared a great number of orthologous genes (1604), accounting for about 47.8 %, 46.7 % and 56.3 % of all genes of XH031^T^, HB2 and DSM 12574, respectively. There were more common genes between XH031^T^ and HB2 (597) than those between XH031^T^ and DSM 12574 (136). COG categories were also compared among the genomes of XH031^T^, HB2 and DSM 12574 (Fig. 4, the pie charts), and the results showed that translation/ribosomal structure (J), energy production and conversion (C), amino acid transport and metabolism (E) and cell wall/membrane/envelope biogenesis (M) were highly conserved among genomes of the three strains. Although HB2 harbors more specific genes (1,093, 31.8 %) than XH031^T^ (1,020, 30.4 %) and DSM 12574 (968, 33.9 %), the functions of the specific genes are categories of general function prediction only (R) and function unknown (S). Meanwhile, XH031^T^ harbors slightly higher proportion of specific genes which belong to inorganic ion transport and metabolism (P), cell wall/membrane/envelope biogenesis (M), transcription (K) and carbohydrate transport and metabolism (G) than the other two strains. Among them, specific genes of transcription (K) in XH031^T^ genome took a larger proportion (9.1 %) than others. More transcriptional regulators in the genome of XH031^T^ might help the strain to develop sophisticated systems to regulate gene expression accurately and thus adapt to the adverse extreme environment.

### Metabolic features oriented with physiological functions

#### Central metabolism

According to KEGG pathway classification (Fig. 1), 1,553 matched genes were predicted to participate in the metabolism of XH031^T^. Among the genes participated in the metabolism, 24.0 % were involved in amino acid metabolism, 19.1 %, 10.6 %, 7.6 %, 7.3 %, 5.7 % and 8.0 % genes were involved in carbohydrate, energy, nucleotide, lipid, enzyme and xenobiotics biodegradation metabolism, respectively. All these metabolism-associated genes in XH031^T^ ensure its survival advantage in harsh marine environment.

For central carbohydrate metabolism, XH031^T^ genome harbors a full set of genes that encoded essential enzymes to carry out Embden-Meyerhof-Parnas pathway. Interestingly, hexokinase is absent, but is replaced by three glucokinases (GL001731, GL003323 and GL003530) in the genome of XH031^T^. In microorganisms, glucokinase and permease genes can offer an alternative or supplemental way for glucose entrying into glycolysis [11]. In addition, all the enzymes involved in tricarboxylic/citricacid cycle (CAC) and hexose monophosphate (HMP) pathways are present. Genes for D-galacturonate and D-glucuronate hydrolysis are also found in the genome (Additional file 2: Table S2). Isocitrate lyase (ICL; GL002375 and GL003473) and malate synthase (MS; GL003472), which are the two key enzymes in glyoxylate cycle, are harbored in the genome of XH031^T^. ICL and MS might be utilized to catalyze pyruvate and acetic acid to synthesize C_4_-dicarboxylate to ensure essential metabolism of the strain. Therefore, glyoxylate cycle as a replenishment pathway might play an important role in the CAC metabolism pathway. These versatile metabolism pathways allow the strain to survive in the extreme environment. However, Entner-Doudoroff (ED) alternate pathway is missing because 2-keto-3-deoxy-6-phosphogluconate aldolas and 6-phosphogluconatedehydratase, the two characteristic enzymes of ED pathway, are absent in XH031^T^.

#### Nutrients metabolisms

Metabolic pathways of diverse monosaccharides, disaccharides and aminosugars were presented in XH031^T^ genome, which revealed the capability of the strain utilizing carbohydrates. For example, *N*-acetylglucosamine-6-phosphate deacetylase (NagA), the key enzyme of utilizing the amino sugar *N*-acetylglucosamine as well as genes of utilizing monosaccharides including ribose, L-fucose and mannose, were predicted in the genome of XH031^T^. Meanwhile, XH031^T^ harbors five predicted glucosidases (2 beta- and 3 alpha-), six xylanases, one alpha-galactosidase and three xylosidases that enable the strain to degrade carbohydrates.

XH031^T^ has more predicted genes of peptidases, lipases and esterases than those of the other four strains (Table 3). Gelatin (collagen) is an important part of the sedimentary organic nitrogen (SON) and high molecular weight dissolved organic nitrogen that is abundant in the deep sea [12, 13]. Extracellular proteases (gelatinase and caseinase) produced by XH031^T^ indicate that this strain might play significant role in degradation of SON. Meantime, 203 peptidases, 6 chitinases and 4 DNases encoding genes were predicted in the genome, agreeing with the experimental results that XH031^T^ could degrade casein, lipids, gelatin and DNA (Table 1).

**Table 3:** The number of predicted enzymes of XH031^T^ and four other strains of genus *Luteimonas*

	*L.abyssi* XH031^T^	*L.huabeiensis* HB2	*L.mephitis* DSM 12574	*Luteimonas* sp*.* J29	*Luteimonas* sp*.* J16
Histidine kinase	47	14	10	10	10
Peptidase	203	45	38	40	38
Glucosidase	5	1	1	1	6
Xylanase	6	2	0	2	2
Xylosidase	3	0	0	1	1
Alpha galactosidase	1	0	0	0	0
Betagalactosidase	0	1	0	1	1
Amylase	3	0	0	0	0
Chitinase	6	1	0	0	0
Other glycosidase	16	3	4	4	4
Lipase	24	3	3	3	3
Esterase	58	4	1	5	5
DNase	4	0	0	0	0

Among the 203 predicted peptidases of XH031^T^, 99 of which have signal peptides. Additionally, there are two chitinases and one amylase with signal peptides. Ninty-nine peptidases with signal peptides of XH031^T^ were allotted to six families in the MEROPS database [14]: serine peptidase (53), metallopeptidase (33), threonine (3), cysteine (3), glutamic acid (1) and aspartic acid peptidases (1), and the remaining were unknown catalytic types. Serine peptidases and metallopeptidases with signal peptides account for 87 % of all extracellular peptidases, coincident with the report that extracellular peptidases of seafloor sedimentary bacteria are mainly serine proteases and metalloproteases families [13]. Various extracellular peptidases harbored by XH031^T^ reveal that the strain might have remarkable ability to degrade a variety of peptides and proteins from its surroundings, which play a major role in the degradation of SON.

XH031^T^ contains all the predicted genes required for fatty acid biosynthesis and oxidation. Polar lipids including phosphatidylethanolamine (PE), phosphatidylglycerol (PG), diphosphatidylglycerol (DPG) and some unknown phospholipid (PL) have been experimentally identified in this strain [10]. In addition, there are four lysophospholipases, five carboxyl esterases, two phospholipases A1, two GDSL, two GDXG family lipases, six thioesterase family proteins and three metallophosphoesterase family proteins encoding genes that have been predicted in the genome of XH031^T^. These enzymes might be used by XH031^T^ to decompose organic matter containing carbon, phosphorus and sulphur.

#### Elements (N, S and Fe) metabolisms

Nitrogen recycling is very important within the marine system and the main flow of nitrogen is from organic nitrogen to nitrate (or nitrite) to ammonium and backward. Two predicted nitrite reductases (GL000686, GL000805) might have the responsibility for the change of nitrite to ammonia in XH031^T^. One nitrite transporter (GL003071) and one putative nitrate/nitrite response responser (GL003071) in the genome of XH031^T^ suggest that this strain might absorb nitrite from the sediment when nitrite is available.

XH031^T^ has one predicted sulfate transporter (GL001999), one predicted sulfate/thiosulfate transporter subunit (GL000105) and the corresponding permease subunit (GL000106), which might transport sulfate ions into the cells. In addition, one subunit of sulfate adenylyltransferase (GL000798) and one adenylylsulfate kinase (GL000799) predicted in the genome might be utilized to convert sulfate to adenylyl sulfate (APS) then to 3'-phosphoadenylyl sulfate (PAPS). PAPS can be catalyzed to sulfite by PAPS reductase. Meanwhile, two sulfite reductase gene clusters (GL000796, GL000797) along with flavoprotein and hemoprotein in XH031^T^ might be used to deoxidize sulfite to hydrogen sulfide (H_2_S). Two predicted phosphoadenosine phosphosulphate reductases (GL000795, GL000798) might take part in the sulfate metabolism. Three predicted cysteine synthases (GL000496, GL000676 and GL000790) from the genome might be utilized to convert H_2_S to cysteine which may enter amino acid or other metabolism pathways.

Iron is one of the mineral elements that are indispensable for microbial growth [15]. It functions mainly in the reduction of ribonucleotides, the transport, storage and activation of oxygen, and other electron transport through a series of electron carriers that span a range of redox potential. The primary form of ferrum (Fe) is the trivalent Fe (III) in organism, and ferric reductases are usually soluble flavin reductases in prokaryotes [16]. Usually, the reduction of ferric iron in organism is very important during cellular iron uptake. XH031^T^ has one predicted ferric reductase (GL000225) and two units of ferredoxin nitrite reductases (GL000686, GL000805), which might catalyze ferric to dissoluble ferrous iron and enter the iron metabolism pathways.

#### Transport systems

Protein secretion plays pivotal roles in regulating the interactions of microbes with their living environments [17]. Six types of secretion system (Type І-VІ), as well as the two-arginine translocation (Tat) and Sec-SRP export systems are present in the genome of XH031^T^. Except for pathogenicity, recent studies indicate that type III secretion system (T3SS) may be used for the formation of biofilm to cope with complicated conditions [18].

Sixteen predicted translocase proteins were found in the genome of XH031^T^ including Tat translocon (TatABCE), Sec translocon (SecABDEFGY), YidC and YajC translocon. In addition, one DNA translocase (GL001427) and three ATP/ADP translocases (GL000181, GL000837 and GL002697) were also found in the genome.

A total of 172 genes were predicted to encode transporter proteins in the genome of XH031^T^, including 85 ATP-binding cassette (ABC) superfamily transporters, 49 TonB-dependent receptors and 38 MFS gene clusters. ABC-type transporters are widespread among microbes and could transport various substrates across intra- and extracellular membranes, including sugars, mono- and oligosaccharides, metabolic products, peptides, cations, toxins, drugs, vitamins and amino acids [19]. Some of the ABC transporters have developed an important function of multidrug resistance (MDR). *Zunongwangia profunda* SM-A87 isolated from deep-sea sediment have been reported to have 40 ABC-type transporters [20]. Both XH031^T^ and SM-A87 verified the report that ABC transporters are very rich in deep-sea microbes [21]. Moreover, three amino acid permeases (AAPs) and four amino acid transporters (AATs) were predicted in XH031^T^. AAPs and AATs may contribute to absorbing amino acids and oligopeptides. Further, molybdate, tungstate, lipopolysaccharides and lipoproteins might be transported through the transport systems of XH031^T^.

In addition, a large number of genes which encode xylose, glucose/galactose, lactose, fucose and arabinose carbohydrate permease were predicted in the genome. Permeases and transporters existing in XH031^T^ might help the strain to uptake nutrients in the intricate ecologic niche of the deep-sea sediment.

As one of the constituents of cell-surface signaling systems (CSS), TonB-dependent outer-membrane proteins make a difference in sensing extracellular signals of bacteria and transmitting them into the cytoplasm [22]. Three TonB protein genes (GL001351, GL002531 and GL003254) and 50 TonB-dependent receptors were found in the genome of XH031^T^, which is in accordance with the previous report that TonB proteins are fewer than TonB receptors [23]. The genome contains 10 predicted TonB-dependent siderophore receptors, which might be involved in the course of iron transporting into cells to be available for metabolic functions [24]. Furthermore, 35 putative outer membrane protein genes were predicted in the genome, and most of them encode peptidoglycan-associated proteins. These features of the secretory system and transporters reveal that XH031^T^ might have a tight relationship with its environments.

#### Substrates utilization

Polysaccharide is one of the main sources of carbon and energy. In the extreme marine environment, polysaccharides can help microorganisms condense organic ingredients, absorb metal ions, and form biofilms [25, 26]. XH031^T^ genome contains 28 predicted glycosyltransferases, of which eight belong to family І and ten belong to family II. Glycosyltransferases can help the strain to synthesize oligosaccharides, disaccharides and polysaccharides [27]. According to the COG database, genes encoding carbohydrate transport and metabolism account for 4.78 % of the whole genome (Fig. 2), which indicates that XH031^T^ has many predicted enzymes involved in degrading oligo- and polysaccharides. The genome also contains three genes encoding exopolysaccharide biosynthesis protein, including one capsular exopolysaccharide family protein, to conduct the synthesis of exopolysaccharides.

XH031^T^ showed many kinds of polysaccharide hydrolysis activities such as amylase, cellulase, and chitinase at low temperature by experiments (Table 1). It had more predicted polysaccharide hydrolysis enzymes than four other strains of genus *Luteimonas* (Table 3). Three annotated amylase genes, six chitinase genes and one cellulase gene were predicted in the genome, which were in agreement with our experimental results that it had the ability to degrade starch, chitin and cellulose (Table 1). The other four strains of genus *Luteimonas* had no amylase and chitinase coding genes, with the exception that *L.huabeiensis* HB2 had just one chitinase coding gene.

Based on COG annotation, XH031^T^ harbors some predicted enzymes to metabolize DNA and RNA, which included tatD DNase family protein (EC 3.1.21), Mg-dependent DNase L and ATP-dependent exonuclease V (alpha and beta subunits). Since DNA is plentiful in seafloor sediments, more than half of them can be degraded rapidly by DNase [28]. The hydrolysate of DNA can provide carbon and phosphorus sources which may participate in the process of biogeochemical cycle. Thus, a variety of extracellular enzymes presented in sedimentary bacteria elucidates their important ecological role in SON degradation. The versatility of metabolic pathways enables the strain to utilize various nutrients much easier in the seafloor sediment.

### Defensive mechanisms

Two L-lactate/malate dehydrogenases, cytochrome bd ubiquinol oxidase subunits I (GL000066), subunits II (GL000067) and cytochrome c oxidase were predicted in the genome of XH031^T^. Acting as the terminal oxidases of the electron transfer chains of many bacteria, the cytochrome oxidases have high oxygen affinity and can impede the toxity of reactive oxygen [29]. The existence of these enzymes in XH031^T^ might help it to survive in the microaerobic and toxic environment of the deep sea. In addition, the strain harbors predicted Superoxide Dismutases (SOD, EC1.15.1.1), which include Fe/Mn family-SOD, Mn/Zn family-SOD, Fe-SOD, Mn-SOD and Cu/Zn SOD. Bacterial SOD nullified the bactericidal activity of O_2_^-^ by changing it into H_2_O_2_, while catalase might be used to dislodge the toxic effects of H_2_O_2_ [30]. As anti-oxidant enzymes, SOD and catalase helped the strain to antagonize oxygen toxicity, which formed a part of defensive mechanism in the adverse environment. However, bacteriorhodopsin and retinal genes involved in photophosphorylation are absent in XH031^T^ genome, consistent with the dark seafloor conditions in which the isolate survived.

XH031^T^ possesses type IV pilus assembly mechanism including a cluster of 18 pili genes. The *pilA* gene encoding the major structure of pili is present in the genome, while *pilR* and *pilS* genes are absent. *PilR* and *pilS* mutants were reported to defense against the infection of phage Cf and be susceptible to Cf infection, respectively [31]. The loss of *pilR* and *pilS* may inhibit the invasion of phage, which provides XH031^T^ survival advantages in such disadvantageous environment.

### Two-component regulatory system (TCRS)

Two-component regulatory system (TCRS), as one of the transmembrane signal transduction mechanisms, is utilized by bacteria to sense and respond to environmental conditions. Typical TCRS are composed of a transmembrane dimeric sensor histidine kinase (HK) and acytoplasmic cognate response regulator (RR) [32–35], which are involved in regulating various biological processes, such as chemotaxis, osmolarity, bacteriolysis and differentiation [36–38]. HKs and other predicted enzymes of XH031^T^ compared with other four strains were shown in Table 3. The number of two-component proteins varies greatly among bacteria. Generally, strains that are more adaptive in their environment usually have larger number of TCRS genes [39]. For example, *Pseudomonas syringae* pv. tomato DC3000 is a widespread bacterial plant pathogen that needs a sophisticated array of TCRS proteins to deal with diverse plant hosts and other adverse environmental conditions, which has been reported to have the largest number of TCRS genes with 69 HKs and 95 RRs [39]. XH031^T^ has 47 HKs and 68 RRs involved in TCRS according to genomic analysis. Among these proteins, 12 predicted proteins contain both the HK and RR domains, each of these different pairs dedicating to unique signals to tackle intricate living conditions.

In TCRS, bacterial signal-transducing protein NtrB and bacterial enhancer-binding protein NtrC are the two important transcription regulator proteins of bacteria [40, 41]. Phosphorylation can be utilized to transfer information between regulator proteins each other. Through phosphorylation relay between regulator proteins, some transcription promoters can be activated to regulate hydrostatic pressure in deep-sea bacteria. For example, σ^54^ promoter, as one of the σ factors, has been found to have a function in pressure-regulated transcription in *Shewanella violacea,* which is a deep-sea piezophilic bacterium [42]. XH031^T^ contains one NtrB (GL003435) and six NtrC (GL000281, GL001446, GL002570, GL002571, GL003435 and GL003436) genes which might play a part in regulating hydrostatic pressure to enable XH031^T^ to live in the deep-sea environment.

### Chemotaxis

A total of 36 ORFs are related to chemotaxis (Additional file 3: Table S3), of which six encode methyl-accepting chemotaxis proteins (MCPs). Fifty ORFs were found to encode chemosensory transducer proteins and 17 ORFs encoded adenylyl cyclase MCPs. All the predicted MCPs in XH031^T^ have transmembrane domains, and some conserved domains of MCPs have been identified. The plasmid achromobacter secretion (PAS) domain was found in three MCPs (GL000612, GL001443 and GL001692). The PAS domain was reported to have been as an aerotaxis receptor to interact with adenylyl cyclase MCP for signal transduction in *E. coli* [43], and the PAS domain predicted in XH031^T^ might contribute to the adaption to microaerobic conditions.

### Enzymes associated with secondary metabolite biosynthesis

Multidomain modular non-ribosomalpeptide synthetase (NRPS) and polyketide synthase (PKS) are the two important enzymes detected in bacteria and fungi to function in biosynthesis of secondary metabolite [44–46]. According to COG database, there are two putative NRPS modules with related protein genes, and 26 polyketide synthase (PKS) gene clusters in XH031^T^ [Additional file 4: Table S4]. The two NRPS predicted in XH031^T^ genome were found to encode PKS, which might involve in putative secondary metabolism pathways. Polyketide is the secondary metabolite including pigment, antibiotic and mycotoxin produced by PKS in microbes or plants [47–50]. In addition, ABC and MFS transporters have the function in synthesizing toxic compounds, such as fungicides and other antimycotic agents [51]. It was reported that MFS transporters can regulate the production of penicillin and heighten the sensibility of *Penicillium chrysogenum* to phenylacetic acid [52]. Meanwhile, four drug resistance transporters and one tetracycline resistance protein (TetA) of MFS were found to be encoded in the genome of XH031^T^, signifying that XH031^T^ might evolve hereditary adaptation to live in toxic environment.

Cytochrome P450s (CP450s) consist of heme-thiolate proteins that ubiquitously distribute in all domains of life and play critical roles in various substrate metabolisms. These substrates include not only endogenous chemicals such as steroids but also xenobiotic complexes including drugs, pesticides and environmental contaminants [53]. XH031^T^ has metabolism related genes of cytochrome P450s, and four of them are glutathione s-transferases which might participate in glutathione metabolism or xenobiotics biodegradation and metabolism [Additional file 4: Table S4]. The existence of CP450s in XH031^T^ might be essential for the synthesis of the primary or secondary metabolites.

### Tolerance to salinity and low temperature

Choline-glycine betaine transporter has been reported to exist in many marine microbes such as *Z. profunda* SM-A87 and *Gramellaforsetii* KT0803 to adapt to salty marine environment [20]. Glycine betaine is a preferential protective solute to regulate osmotic balance in hypersaline environments [54]. And choline as precursor can be converted to glycine betaine by using a two-step oxidation process. XH031^T^ is a halophile that has an optimum growth rate at 0–3 % (w/v) NaCl salinity, with 0–11 % (w/v) NaCl salinity range in nutrient broth (NB; Difco) [10]. One choline-glycine betaine transporter predicted in XH031^T^ genome reveals that the strain might use organic compatible solutes to keep its cellular osmotic balance. In addition, trehalose is a stress metabolite to function in desiccation tolerance, cold resistance and also osmoregulation in bacteria. XH031^T^ contains two predicted trehalose-6-phosphate synthases implying that the strain might have the capability to synthesize trehalose to antagonize adverse conditions.

To maintain the fluidity of membrane is a crucial challenge for the microbes in survival at low temperature. The amount of unsaturated fatty acids can affect the fluidity of the membrane. XH031^T^ harbors three predicted type-І fatty acid desaturase genes, which might dedicate to synthesize unsaturated fatty acids and thus to fit for cold temperature. Traces of unsaturated fatty acids such as C_15: 1_*ω*6c and C_17:1_*ω*8c have been detected in the previous study [10], consistent with the above prediction. Additionally, XH031^T^ harbors seven predicted cold shock proteins (CSPs) including four CspA family proteins, and 12 predicted heat shock proteins (HSPs) with one HslU, DnaK, GrpE, HtpX, HslR respectively and two DnaJ chaperones. Both CSPs and HSPs help the strain to cope with various stresses such as osmotic shock, starvation, heavy metals, ultraviolet radiation or low temperature [55, 56].

### Special structures

As a special structure of Gram-negative bacterium, capsule is a key role in the process of nutrient conservation, adsorption, information recognition, ion interchange, etc. Two capsular polysaccharide biosynthesis proteins predicted in the genome indicate that the strain might have the ability to form capsule.

Flagellum is the most effective cellular structure to conduct taxis (chemotaxis, phototaxis, oxygentaxis or magntotaxis) for bacteria. XH031^T^ has a polar flagellum observed by transmission electron microscope [10], and genome analysis showed that there were 37 genes involved in the assembly of flagellum of XH031^T^. As two transcriptional activator proteins, FlhC and FlhD have been reported to affect cell division in *Erwinia carotovora* subsp. *carotovora* and *E. coli* respectively [57, 58]. FlhC and FlhD were not predicted in XH031^T^ genome. The absence of FlhCD may help bacterial strains keep a stable division rate to respond immediately to environmental changes thus providing competitive advantages [59]. A biofilm is a community of microbes formed by multiple bacterial species which can resist various environmental stresses of ocean environments such as osmotic shock, desiccation, pH shifts or ultraviolet radiation [60]. Six biofilm-forming related genes were found in XH031^T^ genome, showing that XH031^T^ might form biofilm to counteract detrimental surroundings.

### Genetic advantages to adapt to subseafloor sediments

Analysis of the complete genome of XH031^T^ revealed the significant genetic advantages of this strain to adapt to subseafloor sediments. Living at depth exceeding 5,000 m in the deep sea, the strain has to face the two main challenges of low temperature and high hydrostatic pressure. Three type-І fatty acid desaturase genes harbored in XH031^T^ genome may help the strain to keep the membrane fluidity which is a common modulatory mode for cells growing at low temperature. XH031^T^ has been detected to contain 6.5 % of C_16:1_*ω*7c and/or C_16:1_*ω*6c, while *L*. mephitis DSM 12574^T^ only has trace amount (1 %) of it in our previous study [10]. Additionally, TCRS involved in cold signal transduction are contained in XH031^T^ genome manifesting that XH031^T^ has genetic advantage to survive at low temperature. The change of membrane fluidity may affect membrane-associated functions, such as transportation of nutrients. It has been reported that the C_16:1_ fatty acid increases in relative abundance and the C_14:0_ decreases proportionally in marine psychrophilic vibrio during starvation, which results in an increase of membrane fluidity and enables essential nutrients to be transported across cellular membrane [61]. Delong and Yayanos found that the ratio of unsaturated to saturated fatty acids as well as long-chained ployunsaturated fatty acids of a barophilic marine bacterium increase with the increasing of hydrostatic pressure [62, 63], resulting in a raise of membrane fluidity to transport substances across cellular membranes. Therefore, hydrostatic pressure may function in the substances transportation and the mechanism merits further investigation.

Many HSPs and molecular chaperones that may be utilized to antagonize the high hydrostatic pressure were predicted in XH031^T^. As major promoters of HSPs genes, DnaK/DnaJ can be activated under pressure shock, which might help to fold the newly synthesized proteins and prevent misfolding of partially denatured proteins [64, 65], and GrpE may function as a support factor of DnaK/DnaJ [66]. HSPs and other molecular chaperones are responsible for protecting protein or membrane stability, but how they protect cells from high pressure is still unclear.

In addition to low temperature and high pressure, osmotic stress is another property of the deep sea that XH031^T^ must cope with. Extracellular peptidases usually have more aspartic acids, a higher proportion of acidic residues and a lower predicted soelectric point (PI) than the intracellular peptidases. Since low PI and plenty of acidic residues are the two key features of halophilic proteins [67], the extracellular peptidase is more halophilic than the intracellular one. Harboring so many extracellular peptidases (99), XH031^T^ has the ability to live in the saline conditions.

Moreover, various predicted extracellular enzymes (including polysaccharide hydrolytic enzymes, peptidases, etc.) in the genome of XH031^T^ were detected under laboratory conditions. Sedimentary carbohydrate has been reported to be degraded more easily by four enzymes (i.e., lipase, α-amylase, β-glucosidase and peptidase) than by only one enzyme [53]. Compared with other four species of *Luteimonas*, XH031^T^ contains more polysaccharide hydrolytic enzymes, peptidases, lipases and esterases (Table 3), which indicates that XH031^T^ might convert sedimentary bio-polymeric materials into smaller molecules easily and make the absorption of multiple nutrients efficient under the ultra-oligotrophic extreme conditions. At the same time, the oxidation of organic compounds will release maximum amounts of energy through aerobic respiration, while toxic substances such as peroxide or superoxide produced in the aerobic metabolism as by-products can be degraded by catalase, peroxidase or SOD in XH031^T^.

Furthermore, numerous ABC transporters and TonB-dependent receptors were predicted in the genome of XH031^T^. ABC transporters, which are also called traffic ATPases, participate in nutrients uptake and preservation of osmotic homeostasis [68], while TonB-dependent receptors can help large substrate molecules like siderophores and vitamins to be transported into the cell. They may endow the strain a capability to transport various nutrients with high efficiency.

## Conclusions

The whole genome sequence of XH031^T^ exhibited valuable insight in its common and more specific characteristics of deep-sea bacteria, such as numerous ABC-type transporters and extracellular peptidases. In addition, possessing intricate systems (TCRS, CSS, secretion system and substrate transport system etc.) in the genome, the strain has developed hereditary adaptation to thrive in the sediment environment of the SPG. Moreover, comparative analysis with other bacteria of *Luteimonas* demonstrated that the strain had a greater number of exoenzymes to survive in the extreme conditions. Extensive hydrolytic abilities and metabolic versatility unraveled the adaptive strategies and the significant ecological role of the strain in the biogeochemical cycle. Furthermore, the property to export a variety of cold-adapted enzymes provides an extensive application prospect in industry.

## Methods

### Bacterial strain and DNA extraction

XH031^T^ was previously isolated from subseafloor sediment of the South Pacific Gyre during the Integrated Ocean Drilling Program (IODP) Expedition 329, and was identified to be *Luteimonas abyssi* sp. Nov [10]. Genomic DNA was extracted according to standard methods [69]. The quality of the genomic DNA was detected by DNA gel electrophoresis and it showed a pure high molecular weight DNA.

### Screening of extracellular enzymes of strain XH031^T^

Extracellular polysaccharide hydrolases including amylase, cellulase and chitinase were detected on different medium at 28 °C, 16 °C and 4 °C, respectively. These media were marine agar 2216 agar (MA; Becton Dickinson) supplemented with soluble starch (0.2 %, w/v), carboxymethyl cellulose (1 %, w/v) and 1/10 (v/v) chitin colloid (10 %, w/v) [70]. The size of the transparent zone on selective media showed the strength of the enzyme activities. Casein and gelatin cultural media [70] were used to screen extracellular protease. DNase activity was detected by using DNA test agar medium (Qingdao Hope Bio-technology Co., Ltd) according to the manufacturer’s instruction, and 1 M HCl solution was flooded to detect the DNase activity. The lipase screening medium was MA plates supplemented with 0.05 % (V/V) Tween 20, Tween 40 or Tween 80, respectively. When opaque halo appeared on selective medium plate, the strain was positive. Lecithinase medium was used to detect whether the strain can degrade phosphatidylcholine. The medium was MA supplemented with 0.1 liter yolk solution (10 %, v/v) per 1 liter medium [70]. The appearance of milky halo indicated the strain was positive of lecithinase activity. Additionally, H_2_O_2_ was used to detect catalase activity.

### Genome sequencing, annotation and analysis

The draft genome of strain XH031^T^ was sequenced using Illumina HiSeq2000 with a 500-bp paired-end shotgun sequencing which achieved about 42.69-fold coverage. SOAP denovo assembler software was applied to assemble these reads (http://sourceforge.net/projects/soapdenovo2/files/SOAPdenovo2/).The draft genome contained 36 contigs (>500 bp), ranging from 218 bp to 946,316 bp (the N50 and N90 contig sizes were 370,530 bp and 85,099 bp, respectively) which could be assembled into 6 scaffolds. The size of scaffolds was 521 bp to 3,965,915 bp (the N50 and N90 scaffold sizes were all 3,965,915 bp). A total of 3,605 genes were contained in the genome and the total length of genes was 3,549,861 bp which made up 89.01 % of the genome. Six rRNA operons and 51 tRNA operons were predicted by using tRNAscan and RNAmmer software. There were no genomic island and prophage genes according to all these annotation databases. Tandem Repeat (TR) was predicted by using Tandem Repeat Finder (http://tandem.bu.edu/trf/trf.html) [71]. Putative CDSs were detected with Glimmer 3.02 (http://www.cbcb.umd.edu/software/glimmer/) [72, 73]. RNAmmer software (version 1.2) was used to predicted rRNAs (http://www.cbs.dtu.dk/services/RNAmmer/) [74]. tRNAScan-SE (version 1.23) (http://gtrnadb.ucsc.edu/) was used to identify tRNAs [75]. sRNAs were obtained by Rfam (version 10.1) (http://rfam.sanger.ac.uk/) [76]. Function annotation was performed through KEGG (http://www.genome.jp/kegg/) [77–79], COG (http://www.ncbi.nlm.nih.gov/COG/) [80, 81], Swiss-Prot, TrEMBL (http://www.uniprot.org/) and Gene Ontology (GO) (http://www.geneontology.org/) [82], NCBI nonredundant (NR) protein databases (http://www.ncbi.nlm.nih.gov/RefSeq/) [83]. The prediction of signal peptides (SP) was performed using SignalP version 3.0 [84]. Clustal W [85] and Mega 5.0 [86] were used to perform the sequence alignment and phylogenetic analysis.

### Comparative genomics

The complete genome sequences and the general genome features of *L. huabeiensis* HB2, *Luteimonas* sp. J16*, Luteimonas* sp. J29 and *L. mephitis* DSM 12574 were retrieved from NCBI database. The predicted enzymes of the above four strains were obtained from IMG (http://img.jgi.doe.gov/). Proteins from XH031^T^ were compared with these of *L. huabeiensis* HB2 and *L. mephitis* DSM 12574 by using BLASTP with an E-value cutoff of 1e-5. Orthologous proteins to be defined as reciprocal best hit proteins were calculated by the BLAST algorithm, with the minimum 40 % identity and 70 % coverage [59]. Proteins without orthologs were regarded as specific proteins. The COG function category was analyzed by seeking all predicted proteins against COG database according to the BLASTP.

### Nucleotide sequence accession number

This Whole Genome Shotgun project has been deposited at DDBJ/EMBL/GenBank under the accession JUKH00000000. The version described in this paper is version JUKH01000000.

## Additional files

Additional file 1: Table S1. General genomic features of *Luteimonas abyssi* XH031^T^. (DOC 75 kb) Additional file 2: Table S2. ORFs related to chemotaxis in XH031^T^ genome. (DOC 279 kb) Additional file 3: Table S3. The predicted genes to be involved in the general metabolism of *Luteimonas abyssi* XH031^T^. (DOC 108 kb) Additional file 4: Table S4. The genes of secondary metabolite biosynthesis predicted in XH031^T^. (DOC 94 kb)
