# Supplementary material for: Genomic analysis of Luteimonas abyssi XH031T: insights into its adaption to the subseafloor environment of South Pacific Gyre and ecological role in biogeochemical cycle
Source: BMC Genomics. 2015 Dec 21;16:1092. doi: 10.1186/s12864-015-2326-2 (PMC4687298; doi:10.1186/s12864-015-2326-2)
Supplement: Additional file 1: Table S1. — General genomic features of Luteimonas abyssi XH031T. (DOC 75 kb) [file 12864_2015_2326_MOESM1_ESM.doc]

Additional file 1: Table S1 General genomic features of *Luteimonas abyssi* XH031^T^

| **Genome Features** |  |
| --- | --- |
| Genome size (bp) | 3,988,191 |
| G+C content (%) | 69.26 |
| CDSs Number | 3,605 |
| Gene Length (bp) | 3,549,861 |
| Gene Average Length (bp) | 985 |
| Gene Length/Genome (%) | 89.01 |
| GC Content in Gene Region (%) | 69.48 |
| Intergenic Region Length (bp) | 438,330 |
| GC Content in Intergenic Region (%) | 67.48 |
| Intergenic Region Length/Genome (%) | 10.99 |
| Tandem Repeat Number | 272 |
| Tandem Repeat Length (bp) | 29,798 |
| Tandem Repeat Size (bp) | 1-1,923 |
| Tandem Repeat Length/Genome (%) | 0.7472 |
| Minisatellite DNA Number | 191 |
| Microsatellite DNA Number | 21 |
| rRNA operons | 6 |
| tRNA operons | 51 |
| sRNA operons | 0 |
| Genomic Island Number | - |
| Prophage Number | - |
