# Supplementary material for: Genomic analysis of Luteimonas abyssi XH031T: insights into its adaption to the subseafloor environment of South Pacific Gyre and ecological role in biogeochemical cycle
Source: BMC Genomics. 2015 Dec 21;16:1092. doi: 10.1186/s12864-015-2326-2 (PMC4687298; doi:10.1186/s12864-015-2326-2)
Supplement: Additional file 2: Table S2. — ORFs related to chemotaxis in XH031T genome. (DOC 279 kb) [file 12864_2015_2326_MOESM2_ESM.doc]

Additional file 2:

Table S2 ORFs related to chemotaxis in XH031^T^ genome

| **ORF** | **Gene** | **Gene Product** | **Organism with the**  **closest matching**  **sequences** | **E-value** | **Identities** |
| --- | --- | --- | --- | --- | --- |
| GL001513 |  | methyl-accepting chemotaxis sensory transducer | *P.suwonensis* | 0 | 369/730(51%) |
| GL000612 |  | diguanylate phosphodiesterase  with PAS/PAC sensor | *L.dokdonensis* DS-58 | 0 | 421/695(61%) |
| GL001443 |  | diguanylate phosphodiesterase | *X.vesicatoria* | 3e-158 | 289/641(45%) |
| GL001445 |  | diguanylate phosphodiesterase | *P.azotifigens* | 6e-135 | 230/542(42%) |
| GL001951 |  | diguanylate phosphodiesterase | *L.dokdonensis* DS-58 | 0 | 439/701(63%) |
| GL002770 |  | diguanylate phosphodiesterase | *X.vesicatoria* | 3e-158 | 289/641(45%) |
| GL002883 |  | diguanylate phosphodiesterase | *X.campestris pv. cannabis* | 3e-94 | 139/239(58%) |
| GL001499 | *cheA1* | CheA signal transduction histidine kinase | *S.maltophilia* | 0 | 444/623(71%) |
| GL001506 | *cheA2* | CheA signal transduction histidine kinase | *S.maltophilia* | 0 | 437/667(66%) |
| GL001517 | *cheB* | Chemotaxis response regulator protein-glutamate methylesterase | *Stenotrophomonas* sp. RIT309 | 4e-159 | 242/365(66%) |
| GL001516 | *CheD* | chemoreceptor glutamine deamidase CheD | *X. fragariae* | 3e-107 | 151/195(77%) |
| GL001498 | *CheZ* | chemotaxis phosphatase, CheZ | *X.fuscans* | 9e-62 | 120/213(56%) |
| GL000131 | *CheY1* | chemotaxis protein CheY | *L.huabeiensis* | 6e-91 | 135/146(92%) |
| GL000328 | *CheY2* | chemotaxis protein CheY | *L. huabeiensis* | 3e-163 | 273/356(77%) |
| GL001469 | *CheY3* | chemotaxis protein CheY | *L. huabeiensis* | 1e-74 | 112/131(85%) |
| GL001497 | *CheY4* | chemotaxis protein CheY | *S.maltophilia* | 1e-72 | 106/130(82%) |
| GL001505 | *CheY5* | chemotaxis protein CheY | *Xanthomonas citri pv. citri* | 1e-69 | 101/120(84%) |
| GL001631 | *CheY6* | chemotaxis protein CheY | *L. huabeiensis* | 7e-74 | 110/122(90%) |
| GL002527 | *CheY7* | chemotaxis protein CheY | *L. huabeiensis* | 5e-67 | 103/123(84%) |
| GL002571 | *CheY8* | chemotaxis protein CheY | *L. huabeiensis* | 0 | 397/486(82%) |
| GL003339 | *CheY9* | chemotaxis protein CheY | *Luteimonas* sp. J29 | 3e-64 | 100/122(82%) |
| GL003456 | *CheY10* | chemotaxis protein CheY | *L.defluvii* IMMIB APB-9 = DSM 18482 | 1e-166 | 279/568(49%) |
| GL001449 | *CheV* | response regulator CheV | *S*.*maltophilia* | 7e-177 | 257/311(83%) |
| GL002672 |  | two-component sensor histidine kinase | *Azoarcus* sp. KH32C | | 3e-60 | Compositional matrix adjust. | 169/450(38%) | | --- | --- | --- | | 169/450(38%) |
| GL002523 | *CheW1* | chemotaxis protein CheW | *L.dokdonensis* DS-58 | 4e-64 | 98/154(64%) |
| GL001508 | *CheW2* | chemotaxis protein CheW | *M. insulare* | 7e-57 | 86/146(59%) |
| GL000123 |  | methyl-accepting chemotaxis protein | *X. hortorum* | 0 | 456/699(65%) |
| GL001440 |  | methyl-accepting chemotaxis protein | *X. translucens* | 0 | 314/396(79%) |
| GL001510 |  | methyl-accepting chemotaxis protein | *X. axonopodis* | 0 | 461/799(58%) |
| GL001511 |  | methyl-accepting chemotaxis protein | *X. translucens* | 0 | 364/560(65%) |
| GL001513 |  | methyl-accepting chemotaxis protein | *P.suwonensis* | 0 | 369/730(51%) |
| GL002526 |  | methyl-accepting chemotaxis protein | *X. translucen* | 0 | 441/663(67%) |
| GL001515 | *CheR* | MCP methyltransferase, CheR-type | *S.maltophilia* | 4e-120 | 175/240(73%) |
| GL000210 |  | PAS/PAC sensor hybrid histidine kinase | *P. spadix* | 2e-131 | 241/582(41%) |
| GL001443 |  | PAS/PAC sensor-containing diguanylate cyclase/phosphodiesterase | *P.suwonensis* | 0 | 451/910(50%) |
| GL001568 |  | PAS/PAC sensor signal transduction histidine kinase | *C. proteolyticus* | 2e-85 | 171/381(45%) |
