# Supplementary material for: Genomic analysis of Luteimonas abyssi XH031T: insights into its adaption to the subseafloor environment of South Pacific Gyre and ecological role in biogeochemical cycle
Source: BMC Genomics. 2015 Dec 21;16:1092. doi: 10.1186/s12864-015-2326-2 (PMC4687298; doi:10.1186/s12864-015-2326-2)
Supplement: Additional file 3: Table S3. — The predicted genes to be involved in the general metabolism of Luteimonas abyssi XH031T. (DOC 108 kb) [file 12864_2015_2326_MOESM3_ESM.doc]

Additional file 3:

Table S3 The predicted genes to be involved in the general metabolism of *Luteimonas abyssi* XH031^T^

| Genes description | Locus tag |
| --- | --- |
| **Embden-Meyerhof-Parnas pathway** |  |
| Phosphoglucomutase (EC 5.4.2.2) | GL003489 |
| Glucose-6-phosphate isomerase (EC 5.3.1.9) | GL001899  GL002924 |
| 6-Phosphofructokinase (EC 2.7.1.11) | GL000584 |
| Fructose-bisphosphate aldolase (EC 4.1.2.13) | GL000674  GL003197 |
| Triosephosphate isomerase (EC 5.3.1.1) | GL001851 |
| Glyceraldehyde 3-phosphate dehydrogenase (EC 1.2.1.12) | GL000668 |
| Phosphoglycerate kinase (EC 2.7.2.3) | GL000670 |
| Phosphoglycerate mutase (EC 5.4.2.1) | GL002351  GL003411  GL003504 |
| Enolase (EC 4.2.1.11) | GL000926  GL001326  GL001993  GL002934  GL003085 |
| Pyruvate kinase (EC 2.7.1.40) | GL000001  GL000673  GL000767  GL001416  GL002375  GL003473 |
| **Tricarboxylic acid cycle** |  |
| Pyruvate dehydrogenase (EC 1.2.4.1) | GL000313  GL000397  GL001718  GL003340 |
| Dihydrolipoamide acetyltransferase (EC 2.3.1.12) | GL000259 |
| Dihydrolipoamide dehydrogenase (EC 1.8.1.4) | GL000261  GL000910  GL001628 |
| Citrate synthase (EC 2.3.3.1) | GL000639  GL002374  GL002376  GL002568 |
| Aconitate hydratase (EC 4.2.1.3) | GL001526  GL001529  GL002798 |
| Isocitrate dehydrogenase (EC 1.1.1.42) | GL002206  GL003231 |
| 2-Oxoglutarate dehydrogenase (EC 1.2.4.2) | GL000259  GL000261  GL000394  GL000396  GL000397  GL000910  GL001626  GL001627  GL001628 |
| Dihydrolipoamide succinyltransferase (EC 2.3.1.61) | GL001627 |
| Succinate-CoA ligase, alpha subunit (EC 6.2.1.5) | GL002568 |
| Succinate dehydrogenase iron-sulfur protein (EC 1.3.99.1) | GL002102 |
| Fumarate hydratase (EC 4.2.1.2) | GL001620  GL001728 |
| Malate dehydrogenase (EC 1.1.1.37) | GL000127  GL000305  GL000537  GL002206  GL002379 |
| **Fructose and mannose metabolism** |  |
| Xylose isomerase (EC 5.3.1.5) | GL003090 |
| Fructokinase (EC 2.7.1.4) | GL000584  GL000623 |
| Mannose-6-phosphate isomerase (EC 5.3.1.8) | GL000514  GL000789  GL002924 |
| Mannose-1-phosphate guanylyltransferase (EC 2.7.7.22) | GL000514  GL000788  GL000789 |
| **D-ribose utilization** |  |
| Ribokinase (EC 2.7.1.15) | GL000565  GL000623  GL002849 |
| Ribose 5-phosphate isomerase (EC 5.3.1.6) | GL000606 |
| **L-fucose utilization**  L-fucose dehydrogenase | GL001422 |
| L-fucose permease | GL002922 |
| **Xylose utilization** |  |
| Xylose isomerase (EC 5.3.1.5) | GL003090 |
| Xylulokinase (EC 2.7.1.17) | GL003091 |
| Xylanase | GL000125  GL003072  GL003076 |
| **D-galacturonate and D-glucuronate utilization**  Altronate dehydrogenases (EC 1.1.1.58) | GL003083 |
| Glucuronate isomerase (EC 5.3.1.12) | GL003075 |
| Mannonate dehydratase (EC 4.2.1.8) | GL003085 |
| 2-Dehydro-3-deoxygluconokinase (EC 2.7.1.45) | GL000037 |
| 2-Dehydro-3-deoxyphosphogluconate aldolase (EC 4.1.2.14) | GL001734 |
| Gluconate 5-dehydrogenase (EC 1.1.1.69) | GL000053 |
| 4-Deoxy-L-threo-5-hexosulose-uronate ketol-isomerase (EC 5.3.1.17) | GL000052 |
| **N-acetylglucosamine utilization** |  |
| N-acetylglucosamine-6-phosphate deacetylase (EC 3.5.1.25), NagA | GL003535 |
| Chitinase | GL000357  GL000728  GL000905  GL000906  GL002456  GL003604 |
| Hexosaminidase (EC 3.2.1.52) | GL002126  GL003528 |
| N-acylglucosamine 2-epimerase (EC5.1.3.8) | GL002924 |
| **Mannose utilization**  Fructose-1,6-bisphosphatase I (3.1.3.11) | GL003285 |
