# Supplementary material for: Genomic analysis of Luteimonas abyssi XH031T: insights into its adaption to the subseafloor environment of South Pacific Gyre and ecological role in biogeochemical cycle
Source: BMC Genomics. 2015 Dec 21;16:1092. doi: 10.1186/s12864-015-2326-2 (PMC4687298; doi:10.1186/s12864-015-2326-2)
Supplement: Additional file 4: Table S4. — The genes of secondary metabolite biosynthesis predicted in XH031T. (DOC 94 kb) [file 12864_2015_2326_MOESM4_ESM.doc]

Additional file 4:

Table S4 Genes of secondary metabolite biosynthesis predicted in XH031^T^

| Gene predicted | Locus tag |
| --- | --- |
| Non-ribosomal peptide synthetase (NRPS) | GL000852 |
|  | GL001231 |
| Polyketide synthase (PKS) | GL000023 |
|  | GL000170 |
|  | GL000192 |
|  | GL000852 |
|  | GL000853 |
|  | GL001215 |
|  | GL001216 |
|  | GL001223 |
|  | GL001231 |
|  | GL001294 |
|  | GL001630 |
|  | GL001695 |
|  | GL001697 |
|  | GL001852 |
|  | GL002149 |
|  | GL002151 |
|  | GL002152 |
|  | GL002354 |
|  | GL002365 |
|  | GL002649 |
|  | GL002977 |
|  | GL002982 |
|  | GL003349 |
|  | GL003351 |
|  | GL003373 |
|  | GL003453 |
| Drug resistance transporter | GL000917 |
|  | GL000986  GL002047  GL003319 |
| Tetracycline resistance protein, TetA /multidrug resistance protein, MdtG  S-(hydroxymethyl) glutathione dehydrogenase / alcohol dehydrogenase 1.1.1.284 1.1.1.1  Flavoprotein pyridine nucleotide cytochrome reductase | GL000847  GL000023  GL000797 |
| glutathione S-transferase 2.5.1.18 | GL001142  GL002178  GL002373  GL002972 |
